# Dr. Dods on Contorted or Rotated Spine

**Published:** 1824-06-01

**Authors:** 


*Pathological Observations on the Rotated or Contorted Spine, commonly called Lateral Curvature, deduced from Practice. In which are shewn the Causes that produce it; the Reason of its being mistaken for an Incurvation of the Spinal Column; and the Means best adapted to its Prevention and Cure; agreeably to the Principles laid down, and the Author's Experience.* By ANDREW DODS, M.D. Late of Edinburgh, and Surgeon in the Royal Navy. Octavo, pp. 239. London 1824.

WITHIN the short period of twelve months, the pages of this Journal* have contained detailed accounts of two standard works on the subject of *Lateral Curvature of the Spine*, and our attention is now called to a third on the same subject, possessing many claims to our notice. The literary history of every science, and more especially of medicine, shows, that one book (if really read or worth reading) on any given subject, is almost sure to produce others, even although there existed no sufficient grounds for the first parent of the race. In the present case, however, there can be no doubt that ample reasons may be found for the appearance of all the works alluded to, independently of any motives of imitation, emulation, or opposition. In the first place, the disease, to the consideration of which these volumes are devoted, is one which, as well from the various distress produced by it, as from the great difficulty of curing it, must be allowed by all acquainted with it, to be of the very first importance; in the second place, it is a melancholy but undoubted fact, that this disease has amazingly increased in frequency of late years, and now prevails to a most apalling extent; thirdly, it must be admitted that much difference of opinion, and many erroneous notions, both as regards the nature and proper treatment of it, have long existed, and still exist among medical men; and, lastly, it is but doing simple justice to the authors of the works indicated, to declare, that *all* of them have presented important information respecting the matters treated by them. To the merits of the authors of the works formerly reviewed, we hope we have done sufficient justice, in giving in our pages an epitome of their doctrines, with the commendations to which we considered them entitled.

* See Review of Ward in No. 12, and of Shaw in No. 16.

In the present article, it will be our endeavour to mete out to the author of the work before us the same impartial justice. Although, in one respect, the little work of Dr. Dods contains more original pathology than either of the others, its main strength certainly lies in the full and forcible exposition of the *causes* of the disease, as existing in the present fashionable modes of education. Its style is more popular; and, on this account, as well as from the nature of its subject generally, it is much more calculated for the general reader than either the volume of Mr. Ward or Mr. Shaw. In making this statement, however, it is only doing justice to the author to add, that in no one instance does he appear to have forgotten the proper dignity of his rank, or to have gone out of the way to court the attention of extra-professional readers. The book is certainly diffuse, and in some places declamatory; but these blemishes of manner are evidently the consequence of the author's profound conviction of the vast importance of his subject—of a commendable earnestness in the cause of nature, truth, and humanity—and of a pardonable enthusiasm in the support of doctrines which he considers as at once original, and of the greatest moment to the welfare of mankind. Indeed, we are not sure whether these very defects may not be calculated to advance its popularity and increase its usefulness, among that class of readers whom the subject most nearly concerns—viz. the parents of the rising generation of females; and to all of these we most earnestly recommend it, as a work containing information and advice, of which they ought to be neither ignorant nor regardless. It is only by opening the eyes of parents and teachers to the extreme frequency of this disease, and by holding up to them the picture of its miseries, its causes, and the means of prevention, that any rational hope can be entertained of checking its fearful ravages. These expressions, perhaps, may seem unnecessarily strong to such members of the profession as, from particular circumstances, have not been led to see much of this disease; to those, however, who are fully acquainted with its character, and who, like ourselves, have had occasion to deplore, for many years past, the silent inroads it has been making upon the vigour or beauty of one or other of the daughters of almost every family of our acquaintance—our language will not appear unmeasured. In truth, we are sometimes disposed to go so far, on reflecting on this subject, as to doubt whether even the giant malady of our land, *consumption*, is to be considered as more terrible than the one under consideration! Consumption, it is true, is daily and hourly seen decimating the ranks of youth and beauty, and dooming thousands and ten-thousands of its interesting victims to untimely graves—unchecked and almost unopposed by medicine. In this case, however, the period of disease is generally short, and the sufferings of the patient inconsiderable; the malady being chiefly terrible from the desolation and misery it produces in the breasts of surviving friends. Spinal disease, on the other hand, while it rarely kills, is productive of the greatest suffering, (we do not mean *pain)* both physical and moral, to the miserable subjects of it. While still preserving the aspect and the feelings of health, (as often happens) they are hardly capable of enjoying any of the pleasures, or of performing any of the duties, of active and social life: and happy, certainly, would it be for many of them, if, like the victims of consumption, they "might die and be at peace," instead of dragging on a painful and useless existence from year to year, "dying every day they live." It is, truly, at once a melancholy and apalling consideration, how large a proportion of the young ladies of the present day, who are fashionably educated,—whether at boarding schools or under the domestic roof,—are the victims of some degree or other of this affection: and, in reflecting upon this, we cannot help being struck with the humbling lesson which is thereby read to the pride of man. Is, then, all our boasted intellectual cultivation of the present day, and our mental superiority over former times, purchased merely at the price of our physical perfection? Is such the poverty of our common nature as to be unsusceptible of the simultaneous and coequal cultivation of both its parts? Is knowledge a weakness? Is genius a disease?

One thing at least is certain, not only that the bodily vigour of the females of the upper and middle classes of society has been materially injured by the fashionable modes of instruction that have now for many years prevailed,—but that even the general health of the present generation in England, has suffered most grievously from the increased diffusion of education among the people: and it seems to us a question worthy of the gravest and most mature consideration, whether the sum of human happiness, virtue, and social usefulness, has or has not encreased proportionably.

In asserting the so general prevalence of this form of spinal disease, we think it necessary to add that, hitherto, it has almost constantly been overlooked or mistaken by the generality of practitioners, as well as by the patients themselves;—the various symptoms produced by it being considered either as idiopathic affections, or as the consequences of other and more familiar diseases. And it is in respect of the earlier symptoms of this disease, and indeed of its *general* symptoms at every stage, that we consider all the works hitherto published on the subject as particularly defective. Had we space or time, on the present occasion, we think we could sketch a brief outline of the more characteristic features of this malady, as manifested in the state of the general health, even in the earliest stages, which might be useful to our junior brethren; but, perhaps, we shall be considesed as more legitimate members of the ancient craft of criticism by indicating, rather than supplying, the omissions of our authors. The want of such knowledge is sufficiently evinced by the oversights and blunders constantly observed in the practice of those *ontologists* (as Broussais calls them) who look upon diseases as mere collections of symptoms, and recognise no collection as such, that is not set down in the catalogues of Sauvages or Cullen. How frequently, for example, do we meet with weakly women who have been treated—perhaps for years—as the subjects of some chronic inflammation of one or other of the abdominal, pelvic, or thoracic viscera, (according to the site of the pain,) by leeches, blisters, issues, &c. or who have been considered as purely nervous, or simply debilitated; or as labouring under a local disease of the joints or muscles of the extremities; but whose ailments were altogether the consequence, immediate or remote, of this form of spinal disease?

We shall now proceed to give some account of the work before us, confining our attention principally to such parts of it as contain any thing novel in pathology and practice, and passing over such views and doctrines as we have already discussed in our previous articles on the same subject.

In his First Section, entitled "Introductory Observations on the Rotated or Contorted Spine," the author takes a general view of the opinions entertained by preceding and contemporary writers respecting the nature of the disease usually known by the name *Lateral Curvature* of the Spine. After showing that Pott was far from confounding this disease with the carious affection of the vertebræ so well described by him, he notices the views taken of it by Baynton, Lloyd, Wilson, &c. all of whom consider it as consisting of a lateral *yielding* or *giving-way* of the spinal column, in one or more places, from local disease or debility of the yielding part; and then briefly unfolds his own pathology, asserting boldly and unequivocally, in opposition to all former writers, that in this disease THE SPINE IS NOT CURVED AT ALL; or, at least, *is not at all unnaturally curved in its longitudinal direction*.

"I consider it to be an affection totally independent of any disease or diseased action, either in the vertebræ themselves, or in their connecting ligaments or cartilages, and that it is produced, in every instance, by a peculiar affection of the muscles of the back, which affection of the muscles does not, in my opinion, primarily, and necessarily, unnaturally crook or curve the spine in any direction, but ROTATES or TWISTS it in the line of its axis; and that this rotation, or twisting of the spine, is, in itself, sufficient to explain, in the most satisfactory manner, all the phenomena of this important deformity." 30.

By this rotation or twisting of the spine in the line of its axis, there is exhibited to our view a *profile* of the natural curves of the column; and it is the unnatural exposure of these natural curves, our author maintains, that has occasioned the universal mistake respecting the true pathology of the disease.

In his Second Section the author enters, at great length, into the consideration of the remote and immediate causes of the disease. The immediate cause he considers to be an organic affection of the muscles of the back and trunk, produced by the improper exercise of their functions, arising principally from malposition of the body. He prefaces his examination of the various remote causes by an enquiry into the natural functions and healthy action of mucles. These consist essentially in alternate contraction and relaxation, the due and regular performance of both of which is necessary to their healthy condition. Either or both of these functions may be too much or too little performed for the sanity of the parts. The total disorganization of muscular substance from inactivity has been well illustrated by Mr. Shaw, (see our last number;) and is practically confirmed by the experience of our author. He seems, however, to consider a state of prolonged *contraction*, rather than mere inactivity, to be the great agent in disorganizing muscular fibre; and it is especially to the long-protracted contraction of the spinal muscles produced by malposition, that he attributes the origin of the contorted spine.

"Position of whatever part of the body, if long continued, will certainly disorganize its muscles; and it matters not what position may be assumed. It appears to me, however, that this disorganization of a muscle is more the effect of its constant contraction than of its constant relaxation. For example, if we extend one of our limbs and keep it in that position, we shall find that it will be the extensor muscles which will chiefly suffer, that is, they will become permanently contracted, and probably never admit again of being fully relaxed. Whereas, if the limb be retained in the bent position, we shall find that it will be the flexor muscles which will be so affected. While I state this, I admit, at the same time, that a muscle's power of contraction may become much or wholly impaired, from its being kept constantly in a state of relaxation. Yet I am of opinion, that it will bear this state for a much longer period, and with much more ease and safety to the person, than it will do that of contraction." 73.

In applying this fact to the case of the spine, he makes the following observations:

"Man was certainly formed to walk upright, but it was decreed him likewise that he should bend his back; and he who fails to do so shall not go unpunished for his disobedience. It is the want of this simple motion of the body alone, the want of the wholesome and necessary interchange of contraction and relaxation in its muscles, or, in other words, the improper performance of the function of the spinal muscles, that is contorting so many of our young females in the present day; and until the practice ceases of teaching them to keep their bodies so much in the erect position, and the means every where imposed upon them now to effect it be wholly abolished, their numbers will still increase.

"But how much do we see this wholesome and necessary interchange of contraction and relaxation in the muscles of the spine abused, and even denied to children by every possible means! The importunate cry and appalling threats of a mother or tutor, aided by the painful restraint of some favourite instrument, deprive them of it through the long and wearisome day, while the very beds in which they lie down to repose at night are carefully constructed to prevent it." 56.

"The causes, then, of the organic disease of the muscles of the back, which I have said produces contortion of the spine, may be briefly stated to be whatever shall, by retaining the body in the erect or extended position, keep them too much in a state of contraction, and prevent in them the necessary interchange of relaxation; whether this shall be occasioned by the voluntary effort of the child itself, to obey the importunate cry of its mother or governess, *to keep the head up and shoulders back*, or by any of those painful instruments which are so much used now for this purpose." 72.

In proof of the correctness of his views of the general causes of this disease, the author adduces the well-known fact of the comparative immunity of boys generally, and of both boys and girls among the labouring classes, from this deformity. And indeed this proof is quite conclusive; although we find Dr. Harrison, with astonishing simplicity, bringing forward the general straightness of the labourer as a proof that the cause of curvature does *not* exist in the muscles!

These effects of irregular action of muscles from malposition, or too long-continued position of the body, are increased by the pressure on the muscles of the trunk by means of stays, &c. which, like the bandages on a limb, tend directly to produce extenuation of the muscles. There can be no doubt of the truth of this observation; although we have often heard adduced by our fair friends, as a conclusive argument against it, the fact, that their grandmothers were not (by our own showing) distorted, although *they* wore stiffer stays than is now the mode. To this we would answer, that the very different habits and circumstances and constitutions of our less polished ancestors enabled them to resist the ill effects of these unnatural supports, much better than their degenerate posterity. Had they also, like the female youth of the present generation, (in place of being permitted to romp with boys, and enjoy at once the benefit of air and exercise, contented with only a *moderate* acquisition of knowledge,) been trained from infancy to curb the natural action of their muscles, "cabin'd, cribb'd, confined," in boarding schools from morn till night, "deprived of the common air and common use of their own limbs," and perched hour after hour on high stools, or *education chairs*, reading, writing, painting, playing, &c. &c. we apprehend that their "*circum pectus et æs triplex*," in place of giving additional support, would have precipitated their decrepitude.

"The progressive effects produced upon the muscles of the back, from the continued application of the causes mentioned, which is the disorganization spoken of, are, in the first place, debility; secondly, a wasting of their substance; and, lastly, permanent contraction of their fibres.

"If continued position, then, shall disorganize the muscles of the human body in the way I have stated; and if this disorganization of the muscles, when it takes place in those of the back, shall contort the spine in the manner hereafter to be shewn, it becomes a matter of the greatest importance to enquire whether the several modes and customs adopted now, during the education of the female youth of this country, for the prevention of this distressing malady, be not the cause of it: and this, I think, will appear evident, from the following considerations.

"1st. That all the means made use of now, for preserving the figures of young girls, and preventing contortion of their spines, from the cry of those who have the care of them, *to keep themselves erect*, or the common stays, down to the reclining board, or school-room floor itself, tend to keep their bodies in the extended position.

"2dly. That the bodies of children being kept in this manner constantly extended, the extensor muscles of their spines are kept, consequently, in a state of constant contraction, and are seldom or never allowed the interchange of relaxation, which is an indispensable part of their function.

"3dly. That all the muscles of the body being subject to the same laws, and liable to become affected from the same causes, the muscles of the spine will suffer disorganization from continued position, in the same manner as those of other parts of the body.

"4thly. That the effect of disorganization of muscles, is always distortion, to a greater or less degree, of the part or member to which they belong, and which they are destined to move.

"5thly. That contortion of the spine is found to prevail a hundredfold amongst the children who make use of these means of prevention, than it does amongst those who do not. Hence its frequency amongst the children of the rich, and its unfrequency amongst the children of the poor; and hence, also, its frequency amongst girls, and its unfrequency amongst boys, even although of the same family.

"6thly. That contortion of the spine has evidently increased, in proportion as the adoption of these means of prevention have been diffused throughout this country." 80.

In section third the author treats "of the manner in which the rotation or contortion of the spine is produced, and the reason of its being mistaken for an incurvation of the spinal column." It is in keeping in view the natural structure of the spine, and the attachments and action of the various muscles, and in considering the disorganizing effect on these muscles, of the habits above-mentioned, that we find an explanation of these various points. For a full account of these we must refer to the work itself, and content ourselves here with a brief outline of the author's doctrines. This we shall endeavour to give in his own words.

"For the accomplishment of the different motions of the spine, there are many muscles, some to regulate the movement of each individual vertebra, and others that of the whole column; and although its general motions depend upon the *tout ensemble* of these, yet there are some on which the force more particularly falls, and which I consider to be principally concerned in contorting the spine, but I have no doubt many more contribute. The muscles I allude to are what are generally called the *long extensors* of the back, viz. the sacro-lumbalis, and longissimus dorsi, with the quadratus lumborum." 85.

"It is evident, then, that as these muscles are inserted into transverse levers, they become muscles of rotation as well as extension. For example, if the extensor muscles on both sides of the spine be thrown into equal action at the same moment, the motion produced will be direct extension of the column; but if they be thrown into action only on one side, the spine, although made to incline in the direction of the muscles' forces to that side, will be moved by means of the rotatory movement of the vertebræ."

"If the extensors of the spine, then, from being inserted into transverse levers (the transverse processes of the vertebræ,) be constituted muscles of rotation, when they act on one side only, it will follow, that should disorganization, producing contraction, take place in those of one side, the effect of this contraction, by its overbalancing the action of their fellows on the opposite side, will be to rotate the vertebræ to which they are attached, and destined to move; and, consequently, to produce rotation or contortion of the column, and not curvature." 88.

The obvious result of this rotation will be, as already said, to bring the natural flexures of the spine, which in the sound condition of the body are concealed, into view in the back; and it is merely these natural flexures thus unnaturally exposed, according to our author, which have hitherto been universally considered as morbid flexures of the vertebral column. The same rotation, it is said, explains every variety of alteration of shape in the bony parietes of the chest, so conspicuous in even the slighter degrees of this deformity. These original pathological views of the author are at once strikingly illustrated and confirmed by the account of the circumstances which first led him to adopt them.

"Having been induced, of late years, to apply myself more particularly to the investigation of this interesting deformity, and being previously convinced, in my own mind, that it depended more upon muscular contraction, brought on by the fashionable modes and customs of the day, than upon structural disease of the vertebræ, I was led to the adoption of friction for its cure; and in order that I might give it the fairest trial possible, I was induced to become the operator myself.

"During the course of my operations upon several patients, I was struck in all of them (for they were all contorted to the right side) with a considerable bony hardness and projection on the left side of the loins, raised nearly to a level with the spinous processes; and this I found to be the case in the patients whose spines exhibited little or no apparent curvature in the loins, as well as in those in whom the apparent curvature was very great. Being led to investigate this anomaly, I redoubled my exertions to discover the cause; and I found, after the muscles had been relaxed by friction (for they were in every case extremely rigid), that the bony projection was the transverse processes of the vertebræ of the loins, which I could now as distinctly feel and count as the spinous. From this circumstance, I was led to examine whether I could feel the transverse processes of the same vertebræ on the opposite side, but without effect, for they appeared to have sunk inwards, completely out of reach. Having satisfied myself of these facts, I then reasoned in the following manner.

"If these distortions of the spine, as it is generally supposed, depended upon a direct lateral curvature of the column, the transverse processes, although they would be, in this case, separated from each other on the one side, and approximated on the other, yet would they not be altered in their transversity, with respect to the body, and consequently ought to be as easily discovered and felt on the right side as on the left, but which I found was not the case. I then asked myself what movement of the vertebræ would bring their transverse processes to be so prominent, and so distinctly felt on the one side, while they were totally out of reach on the other, and I concluded it must be their ROTATION." 102.

For further pathological details on this point, we must refer to the work itself, expressing our full assent to the truth of the principles maintained by him. A most unsuspected and unequivocal testimony of their truth is furnished by Mr. SHAW in several passages of his excellent work, where the effects of the *rotation* contended for by Dr. Dods, are clearly and explicitly described, although without any intimation on the part of the author, of their dependence on this particular pathological state. To the merit of this discovery Dr. Dods seems exclusively entitled. See, for instance, pages 69, 113, 117 of Mr. Shaw's work.

In his fourth and last section, Dr. Dods treats "of the prevention and Cure of the Rotated or Contorted Spine." Under the head of prevention, he once more passes in review the whole system of fashionable education, and utterly proscribes every plan and all sorts of apparatus adopted with the view of keeping the body straight, but with the direct effect of producing distortion. In this list of proscription he includes—position, education chairs,—stays,—backboards,—stocks for the feet,—inclined planes,—lying on the floor, &c. &c.; the only practice that escapes condemnation being *drilling*, and this is considered safe only where the others are *not* enforced.

"When we come to survey this long list of instruments, and the means so universally used now for the fashionable man's children, we cease to be surprised at their bodies being so frequently and miserably deformed, and only wonder how it is that so many escape this calamity.

"It affords us a striking example, I think, of the wonderful efforts of nature to resist the effects of such pernicious instruments; and I consider those children who are reared now to maturity under their use, without being contorted in their spines, only as so many miraculous instances of escape." 150.

In place of all this machinery, and care, and artificial restraint, the author recommends that the plain and simple dictates of nature should be followed in the education of children, justly believing with the poet, that

"God never made his work for man to mend."

"To prevent children from being mutilated by this cruel deformity, let those who have the care of them cease to restrain the position of their bodies in any manner whatever. Let that *attention du corps*, that mania, for it deserves no other name, for training them to sit constantly erect, be wholly laid aside: let the cry of *hold your head up, and keep your shoulders back*, be as seldom heard in the chambers of the splendid mansion, as it is in the lowly cottage; and let all those vile instruments and means so much now in use amongst children which I have mentioned, and others which may have escaped me, no longer find a place in the dwellings of the wealthy. Let children, while nature is yet maturing their tender bodies, be wholly unfettered; let no stays (for they require none) be imposed upon them, neither let them be trained to the constant observance of any particular position, but let them have a comfortable chair or seat, with a back to it, such as I have described, to sit down upon when they feel disposed, and let them lean to the back of it, for it is beneficial that they should do so. And, should their frames be delicate, let them be indulged with a sofa when they are inclined to rest their wearied bodies; but let them not be stretched out upon it in the manner they now are, upon an inclined plane, for it will only increase their debility: but let them lie with their heads raised, and their bodies and spines gently bent, or in whatever other position nature shall instinctively point out; for, rest assured, it is the only way to relax their wearied and contracted muscles, and recruit their exhausted strength. Let them not be penned up in the house all day, trusting only to the exercise which their muscles and bodies shall receive at the command of a drill-sergeant, to preserve them in health, and prevent them from becoming contorted in their spines; but let them sally forth, two or three times a-day, to breathe the open air, and exercise, with vigour and activity, their stiffened limbs; and when they do so, let them not forget that their spines have joints, and muscles to be moved and exercised as well as their limbs. Let them be permitted to toss a ball, or some such thing, and pick it up from the ground when it falls, that their spines may be used in the manner that nature intended. Let their bodies be bent forwards and extended, and extended and bent forwards again, for it is by this simple motion of their bodies alone, that their spines will be preserved in health and strength, and retain their symmetrical and natural form; the neglect, or rather the denial and prevention of which, is the principal, nay, I would say, the only cause of all this deformity now in our land. And, when they return home from such an innocent and healthful sport, let not its wholesome and sanative effects be done away with, by placing them immediately upon a music stool, or education chair, but let them sit down to rest themselves, for half an hour or longer, if it shall be required, upon a comfortable seat, in the manner I have recommended, to recruit the muscular strength and energy which have been expended in their little sportive exercise, when they will again return to their scholastic duties, cheered in their minds, and refreshed in their bodies." 156.

For the details of the plan of cure, we must refer to the original. The disease being the consequence of muscular contraction, the cure is to be effected by producing relaxation of the contracted parts, in the first place; and then, by proper exercise, restoring them to a requisite degree of tone and activity. As might be expected, Dr. Dods condemns all mechanical means and instruments in every stage of the complaint,—with the exception of friction and manipulation, and a simple contrivance to exercise the muscles of the spine. He appears to entertain a horror of all kinds of instruments; and we have reason to believe, that this horror originated from observing the ill effects of these, on a large scale, in a spot long celebrated for their employment as the chief means of cure in such complaints.

The following paragraph contains a brief summary of the author's mode of conducting the cure, and is all that our limits will permit us to extract.

"My patients being provided with, and dressed in flannel dressing gowns, open to the back, and petticoats, I desire them to lie down upon their faces on the friction bed, in the manner described. I then besmear the back well with the prepared oil mentioned, and make use of strong frictions to all the muscles of the spine, but particularly to those which are most contracted; and at the same time I use manipulation, at intervals, to the displaced bones. This process I continue about twenty minutes or half an hour at a time, and repeat it twice, and in some cases, three times a week. If the friction be applied in the evening, the patients are desired to go immediately to bed, in which I order them to lie for some time on their backs, with their heads and shoulders raised a little, and their bodies gently bent, in order that the muscles of the spine may be kept in a state of relaxation, which such a position of the body admits of, and which is quite agreeably to nature's laws. If the friction be applied during the day, instead of sending my patients to bed, I desire them to lie down on their backs upon the relaxing couch, for an hour or so before they dress themselves, and afterwards to resume the couch. This couch they continue to occupy daily, instead of sitting up upon a chair. But as I do not confine my patients constantly to this position, so I direct that they get up frequently, and exercise the muscles of their spines for a quarter of an hour, or twenty minutes at a time, by means of the shoot and balls described, and to resume their couch again immediately after the exercise. I allow my patients likewise to rise to their several meals, and to seat themselves in a chair, such as I have recommended; but if it pleases them to have one with arms, I have no objection to it. I do not allow them, however, to sit up more than half an hour at each meal, when they again return to their couches." 221.

We must here take our leave of Dr. Dods, earnestly recommending his little work not merely to the members of the profession, but to all parents and guardians, and indeed to every one who is at all interested in the education of youth, or in the welfare of the rising generation. We congratulate the public on the prospect now opened to it, of seeing rescued from the rude hands of empirical machinists and ignorant rubbers, a class of diseases as distressing in their nature, and as much requiring for their treatment and cure, a scientific knowledge and application of the principles of medicine, as any in the range of nosology.

*P. S.* Dr. Jarrold's work on the Spine reached us just as the above article was closed. We hope to give some account of it in our next number, as it appears to us to contain some important information.—*Rev.*

